# Semirigid Fiberglass Casting for the Early Management of Clubfoot: A Single-Center Experience

**DOI:** 10.7759/cureus.22683

**Published:** 2022-02-28

**Authors:** Brendan Williams, Jorge N Gil, Samuel Oduwole, Laurel C Blakemore

**Affiliations:** 1 Orthopaedics, Children's Hospital of Philadelphia, Philadelphia, USA; 2 Orthopaedics and Rehabilitation, University of Florida, Gainesville, USA; 3 Orthopaedics, Frank H. Netter MD School of Medicine, New Haven, USA; 4 Orthopaedics, Pediatric Specialists of Virginia, Fairfax, USA; 5 Orthopaedics, Children's National Medical Center, Washington, USA

**Keywords:** ponseti casting, semirigid fiberglass casting, casting material, ctev, clubfoot

## Abstract

Background

Semirigid fiberglass (SRF) is an alternative material to plaster of Paris (POP) for idiopathic clubfoot casting in the Ponseti method. The purpose of this study was to evaluate early clinical outcomes in a series of idiopathic clubfoot patients treated with SRF at a single institution and to compare these findings to historical norms with POP casting present in the literature.

Methods

A series of idiopathic clubfoot patients managed exclusively with SRF in the Ponseti method was identified. Treatment efficacy was evaluated by number of casts, change in Pirani score, frequency of treatment-related complications, and frequency of surgery other than tenotomy. A comprehensive literature review was used for comparative historical norms.

Results

The study included 34 feet in 26 patients. Pirani score was 4.7±1.3 at presentation and 1.9±1.4 at the end of casting, representing a score change of 2.8±1.3 with SRF. Initial correction was obtained with 6.9±1.4 casts. Treatment-related complications occurred in six treated feet (17.6%) including 13 cast slippages in five feet and one cast-related thigh abrasion. A total of 25 (73.5%) feet underwent tenotomy. Two feet required an additional surgical procedure.

Conclusion

Clubfoot patients treated with SRF demonstrated acceptable deformity correction following Ponseti-style casting. The quantitative clinical outcomes evaluated appeared similar to norms using POP present in the literature. The findings of this study support SRF as a viable alternative to plaster casting for clubfoot correction utilizing the Ponseti method. As such, further investigation for rigorous comparative assessment is warranted.

## Introduction

Congenital talipes equinovarus (CTEV), known more commonly as clubfoot, is an idiopathic deformity affecting approximately 1 in 1,000 births [1]. The Ponseti method, consisting of serial manipulation, long leg casting using plaster of Paris (POP), and tendoachilles tenotomy if needed has become the standard of care for early treatment of clubfoot as well as relapses. This technique has high rates of reported success [1-5].

Ponseti method of clubfoot casting has traditionally been practiced utilizing POP casts, and its efficacy has been well documented in the literature [1-10]. Semirigid fiberglass (SRF) casting has been described as an alternative to plaster casting, with prior work suggestive of benefits of higher parent satisfaction and simpler cast removal at the convenience of the provider and parent [4,11]. While some centers have moved to SRF for Ponseti casting material, other providers still feel that POP demonstrates superior moldability and hence superior clinical outcomes [12]. There remains a sentiment among some parents and providers that SRF is inferior to POP casting despite mixed results in head-to-head material comparisons [4,11].

Although recent work [7,11] has suggested promising results and positive provider and parent experiences using SRF casting for clubfoot, the evidence base exploring the efficacy or equivalency of SRF remains sparse. As a result, many providers are still reluctant to use SRF casting for clubfoot due to the tradition of plaster casting and its moldability characteristics. In order to further this clinical debate, we retrospectively evaluated a series of idiopathic clubfoot patients managed using SRF at a single center using commonly reported clubfoot treatment-related outcome measures. These data will facilitate comparison of treatment efficacy with established norms in the literature using POP casting. We hypothesized that outcomes using SRF would be similar to historical norms and that frequency of cast-related complications would be less.

## Materials and methods

Study design and setting

A retrospective review was performed to identify a series of idiopathic clubfoot patients managed with SRF using the Ponseti method at a single institution. The study was conducted at a single center identifying patients treated between January 2014 and December 2016. The start of this study period coincided with the introduction of exclusive use of SRF in clubfoot treatment at our institution. Approval was obtained from the Institutional Review Board at our university.

Clinical management

All patients were managed with serial casting by Ponseti technique by two fellowship-trained pediatric orthopedic surgeons. Pirani scoring was used as a standardized measure for outcome assessment [13]. Scoring was facilitated by placement of instructional posters at clinical workstations to enhance scoring reliability. The end of the initial casting period and the need for heel cord tenotomy or additional surgical procedures was at the discretion of the attending provider.

Participants

Eligible subjects were identified through database searching of patients with associated diagnosis and procedural codes for clubfoot. Records were then screened to identify patients with idiopathic clubfoot. Exclusion criteria were as follows: late presentation (>1 year old), initial treatment (conservative or surgical) at another facility, treatment at any point with plaster casting, non-idiopathic clubfoot (congenital myotonic dystrophy, myelomeningocele or amniotic band syndrome), or missing clinical documentation (e.g., no Pirani score completed).

Data collection and study variables

Medical records of patients meeting inclusion criteria were then reviewed in detail to gather demographic, treatment, and outcome data. Demographic information included age at presentation (continuous), gender (dichotomous), and laterality (dichotomous). Clinical records were reviewed from initial presentation until the initiation of boots and bars to identify the total number of casts required in treatment (discrete) and the Pirani score at each clinical encounter (discrete). The change in Pirani score was then calculated for each foot from initial presentation until the initiation of boots and bars. The occurrence and frequency of any treatment-related complications including cast slippage, skin irritation, pressure injury, or other issues requiring an unplanned visit were noted. Medical records were reviewed to determine whether tenotomy was required as well as any additional surgical interventions (e.g., posteromedial release).

Statistical methods

Descriptive statistics (mean ± standard deviation or percentage) were used to report demographic and treatment-related variables for narrative, subjective comparison to literature-based norms within the discussion. Continuous variables were expressed as means with standard deviation. Statistical analyses were performed using JMP® PRO Version 13.0 (SAS Institute, Cary, NC).

## Results

Patient identification and demographics

A total of 170 patients were identified via diagnosis and procedural codes related to clubfoot during the study period. Exclusions were made for the following criteria: age not meeting inclusion criteria (4), non-idiopathic clubfoot (83), prior treatment at other center and/or by provider not using SRF casting (29), incomplete clinical documentation (25), and did not complete casting treatment at our center (3). After these exclusions, 34 feet in 26 patients were identified meeting study criteria for analysis.

The mean age at presentation of patients was 25.1±22.1 days (range: 4-85 days). The majority (69.2%) of patients were male. Diagnosis was unilateral in 18 patients (72.2% left) and bilateral in 8. The mean Pirani score for each clubfoot at initial encounter was 4.7 ±1.3.

Clinical outcomes

Analyzing by foot, the mean Pirani score was 4.7±1.3 at presentation and 1.9±1.4 at the end of casting. The mean change in Pirani score was 2.8±1.3. Correction was obtained after 6.9±1.4 casts. Twenty-five (73.5%) feet underwent additional percutaneous Achilles tenotomy at the end of cast management.

Of the 34 feet, two feet required an additional surgical procedure, both of which underwent posteromedial release. Treatment-related complications occurred in six (17.6%) feet, which included 13 cast slippages in five feet and a single cast-related thigh abrasion.

## Discussion

Concerns exist among some in the pediatric orthopedic community regarding the moldability and deformity correction efficacy of SRF as compared to POP. However, existing literature evaluating the use of SRF in the Ponseti method suggests favorable patient outcomes and high caregiver satisfaction with this material [7,11,14,15]. While the first study to directly compare the efficacy of Ponseti casting with these two materials suggested superior results with POP [4,16], two more recent comparative studies have indicated comparable outcomes with these materials [11,17]. Monforte et al. found a similar complication rate between the two materials and even reported a shorter duration of casting time for patients in the SRF group. As such, we sought to broaden the evidence base to assess the generalizability of these findings by exploring our center’s outcomes with SRF using a variety of established outcome measures for clubfoot: (1) number of casts required to achieve adequate correction, (2) change in a standardized measure over the casting period (e.g. Pirani score), (3) rate of tenotomy utilization, (4) frequency of surgery other than tenotomy, and (5) frequency of complications. The primary objective of this study was to quantify these measures in a series of patients treated at our center with SRF in order to compare these values to historical norms in patients treated with plaster. We identified that the number of castings, rates of Achilles tenotomy, need for additional surgical procedures, and complication rates were within the range of POP casting in the existing literature [4,14,16,18,19]. Successful deformity correction was achieved without increased risk of complication or need for surgical intervention.

Number of casts utilized

The mean number of plaster casts used to achieve successful correction of clubfoot deformity varies in the literature, ranging from 3.8 to 7.3 required casts [11,16,20-22]. Initial Pirani score and age are the two most influential factors contributing to the number of casts used for correction and can be used to explain such a large variability in regard to quantity of casts used for clubfoot correction [23]. Our results, in keeping with these findings, demonstrated that high Pirani scores at presentation are associated with a higher number of casts. In our cohort, the mean number of casts utilized for clubfoot correction was approximately 7, suggesting a corrective ability within the range of POP casting norms.

Change in Pirani score

The Pirani score [13] was developed as a means to quantify the severity of clubfoot correction in order to monitor treatment effect. Prior to treatment, the range of Pirani scores in children treated for clubfoot is 3.8-5.8 [7,8,11,21,22,24-26]. The literature demonstrates that there is large variability with respect to Pirani scores post-Ponseti management, ranging from 0.5 to 3.49 [14,22,27]. Children treated at our center had a mean initial Pirani score of 4.7 and a mean post-treatment score of 1.9. This represents a Pirani correction score of 2.8 achieved with casting, which is comparable to plaster-based correction scores and is within the range of reported post-treatment scores.

Additional surgical procedures

Achilles tenotomy remains a critical aspect of the Ponseti method in patients with residual equinus deformity after casting. Although utilization of this procedure alone does not necessarily represent treatment failure, it does serve as an indicator of the severity of clubfoot deformity that is being addressed in a given cohort. Numerous studies examine the necessity of percutaneous Achilles tenotomy and reveal a range of 60% to 99.1% of patients undergoing serial casting ultimately require tenotomy [7,9,11,16,21]. Our patients underwent Achilles lengthening at a rate of 73.5%. Thus, the rate of tendoachilles lengthening in this cohort appears to be comparable to POP cohorts.

Surgical intervention beyond Achilles tenotomy is uncommonly required early in the management of idiopathic CTEV. Documented rates are as low as 2-3% after serial plaster casting [2,16]. However, some cohorts have required repeat tenotomy in up to 8-9% [28]. In our cohort, the rate of additional surgery was 6%. Both failed feet required posteromedial releases. Whether or not this represents a true difference in treatment failure is uncertain as there is limited documentation in the literature, and our study sample is small. However, these findings indicate that stubborn and severe cases of clubfoot may demand further surgical intervention irrespective of what casting material is used.

Treatment-related complications

Treatment-related complication reporting in the initial management of clubfoot is poorly standardized and varied, making cross-study comparison somewhat difficult. Complications may be due to cast application technique, duration of application, and cast removal. These complications are reported in varied frequency across the reviewed literature [4,14,16,18,19]. Soft tissue complications and cast slippage in our study was 2.9% and 17.6%, respectively. Based on the available literature for comparison summarized in Table 1, while casting-related soft tissue injury appears less frequent with SRF than historical figures, cast slippage may have occurred at higher rates in our cohort than POP Ponseti casting.

**Table 1:** Summary of existing clubfoot treatment outcomes reported in the literature. Data that were not reported or not calculable based on findings reported in the manuscript are indicated by "-". Some means were estimated based on the data provided by the study and indicated as such. POP, plaster of Paris; SRF, semirigid fiberglass

Author (Year)	Cast Material	Feet (Children)	Pirani Score at Start of Treatment, ±SD (Range) if Reported	Pirani Score at End of Treatment, ±SD (Range) if Reported	Mean # of Casts to Correction, ±SD (Range) if Reported	Tenotomy Required (%)	Other Surgical Procedure Required: Feet (Children); (%) of Feet	Reported Complications: Feet (Children); % of Feet
Scher et al. (2004) [29]	POP	50 (35)	-	-	5.4 (estimated mean)	72%	-	-
Dyer and Davis (2006) [21]	POP	70 (47)	4.6	-	5.31 (2-9)	60%	-	-
Brewster et al. (2008) [7]	SRF	80 (51)	5.5 (3-6)	2.5 (0.5-3)	10	75%	-	Relapse/recurrence: 5(4); 6.25%; cast slippage: 1 (1); 1.25%; minor skin irritation: -
Pittner et al. (2008) [4]	POP	23 (18)	-	-	5.2	87%	Other surgical procedures: 0	Cast slippage - (6); minor skin irritation - (5) (not separated by group)
	SRF	16 (13)	-	-	6.1	87.50%	Posteromedial release: 2 (1); 12.5%	
Jawadi (2010) [16]	POP	235 (175)	5.8 (5.5-6)	0.5 (0-1)	5.2	99.10%	Posteromedial release: 4 (-); 1.7%	Relapse/recurrence: 48 (34); 20.4%; minor complications: 18 (14); 7.65%
Pulak and Swamy (2012) [18]	POP	53 (40)	5.6	-	4.9	94.30%	-	Excoriation of the skin: 7 (-); 13.2%; relapse/recurrence: two cases
Agarwal and Gupta (2014) [30]	POP	442 (297)	4.8 (1-6)	-	7 (2-18)	100%	-	-
Hui et al. (2014) [11]	POP	18 (12)	4.9 (3-6)	-	4.4 ± 1.6	78%	Need for surgery after casting (including posterior release, posteromedial release, tibialis anterior tendon transfer, and tibialis posterior recession): -(2); -	Deformity relapse and need for repeat Ponseti casting
	SRF	26 (18)	5.3 (2-6)	-	5.7 ± 2.8	58%	Need for surgery after casting (including posterior release, posteromedial release, tibialis anterior tendon transfer, and tibialis posterior recession): -(5); -	Deformity relapse and need for repeat Ponseti casting
Aydin et al. (2015) [14]	POP	116 (101)	5.28 ± 0.79	3.32 ± 0.96	3.8	81.90%	-	Skin scratches due to oscillating saw or abrasions: 14; cast slippage: 9
	SRF	113 (95)	5.47 ± 0.92	3.49 ± 1.12	3	84.90%	-	Skin scratches or abrasions: 8; heel ulcerations: 2; cast slippage: 3
Elgohary and Absulaad (2015) [28]	POP	34 (2)	5.17 ± 0.62 (4-6)	0.49 ± 0.42 (0-1)	4.88 ± 0.88	91.20%	Re-tenotomy: 3 (-); 8.8%	Relapse/recurrence: 5 (-); 14.75%
	POP accelerated	32 (21)	5.13 ± 0.61	0.52 ± 0.38	5.15 ± 0.72	93.80%	Re-tenotomy: 3 (-); 9.4%	Relapse/recurrence: 5 (-); 15.6%
								"No major complications" in either group
Smythe et al. (2016) [22]	POP	268 (173)	3.8 ± 1.15	0.80 ± 0.56	7.27	78.90%	-	-
Ayehualem et al. (2019) [19]	POP	424 (287)	5.2 (estimated mean)	-	5.54 (1.63)	76%	-	Premature cast removal due to swelling: 10; knee flexion contracture: 1; dorsal foot wound: 2
Monforte et al. (2021) [17]	POP	68 (42)	4.6	1.17	5.2	95.60%	0	Relapse: - (4); -
	SRF	68 (43)	4.5	1.23	4.2	92.60%	0	Relapse: - (4); -

Limitations

Inherent to this study’s design are multiple limitations. As a retrospective review, the capture of treatment-related complications, particularly minor complications, is limited by the quality of provider documentation. Second, based on our study criteria, we sought to only evaluate idiopathic clubfoot patients treated primarily with SRF, and thus the generalizability of the reported outcomes may vary when applied to a broader set of clubfoot patients. Finally, although the Ponseti method is a well-described technique, there exists some interprovider variability in cast application (e.g. padding, molding, positioning) that may contribute to differential treatment success in centers with higher volume. This study examined patients managed by two providers for whom no data exists in a comparable cohort of patients managed with POP casting.

## Conclusions

This case series indicates that standard measures of treatment-related success for clubfoot patients managed with SRF are comparable to documented norms in plaster casting while skin-related complications may be less frequent. These results, coupled with theoretical patient, provider, and caregiver benefits not measured by these outcomes suggest SRF could be considered as a treatment alternative in the management of idiopathic clubfoot by Pirani casting. Based on these findings, additional prospective studies comparing patient outcomes and examining longer-term treatment success for both materials are warranted.
